# The impact of COVID-related perceived stress and social support on generalized anxiety and major depressive disorders: moderating effects of pre-pandemic mental disorders

**DOI:** 10.1186/s12991-022-00385-3

**Published:** 2022-02-14

**Authors:** Anna Monistrol-Mula, Mireia Felez-Nobrega, Joan Domènech-Abella, Philippe Mortier, Paula Cristóbal-Narváez, Gemma Vilagut, Beatriz Olaya, Montse Ferrer, Aina Gabarrell-Pascuet, Jordi Alonso, Josep Maria Haro

**Affiliations:** 1 Research, Teaching and Innovation Unit, Parc Sanitari Sant Joan de Déu, Sant Boi de Llobregat, Barcelona Spain; 2 Centre for Biomedical Research on Mental Health (CIBERSAM), Madrid, Spain; 3 Department of Sociology, Universitat de Barcelona, Barcelona, Spain; 4 Health Services Research Unit, Hospital del Mar Medical Research Institute (IMIM), Barcelona, Spain; 5 Instituto de Salud Carlos III, Centro de Investigación Biomédica en Red de Epidemiología y Salud Pública (CIBERESP), Madrid, Spain; 6 Department of Life and Health Sciences, Pompeu Fabra University (UPF), Barcelona, Spain; 7 Departament de Medicine, Universitat de Barcelona, Barcelona, Spain

**Keywords:** Affective disorders, SARS-Cov2, Psychiatric disorders, COVID-stress syndrome, Social determinants

## Abstract

**Background:**

We assessed the moderating effect of pre-pandemic mental disorders on the association of COVID-related perceived stress and social support with mental health.

**Methods:**

A nationally representative sample of 3500 Spanish adults was interviewed in June 2020 (mean age 49.25 years, ± 15.64; 51.50% females). Mental health included Generalized Anxiety Disorders (GAD; GAD-7, cut-off point of ≥ 10), Major Depressive Disorders (MDD; PHQ-8, cut-off point of ≥ 10) and the comorbid form (those screening positive for GAD and MDD). COVID-related stress was assessed using an adapted version of the Peri Life Events Scale, and social support using the Oslo Social Support Scale. Logistic regression models were used to assess if COVID-related stress and social support were related to mental health outcomes and interactions were conducted to examine whether these relationships differed according to the presence of pre-pandemic mental disorders.

**Results:**

Higher COVID-related stress was associated with a higher risk of lower mental health. The association between COVID-related stress with GAD and MDD was significantly moderated by pre-pandemic mental disorders, except for comorbid GAD + MDD. Higher levels of social support were linked to better mental health. Only the association between social support and GAD was significantly moderated by pre-pandemic mental disorders. That is, for those without pre-pandemic mental disorders, higher levels of social support decreased the odds of GAD, while minor decreases were observed in those with pre-pandemic mental disorders.

**Conclusions:**

The impact of COVID-related stress and social support on specific indicators of mental health may vary depending on the existence of a previous mental disorder.

## Background

While societies continue to struggle to slow down the transmission of the SARS-Cov-2 (severe acute respiratory syndrome coronavirus 2), the COVID-19 (coronavirus infectious disease 19) pandemic is expected to have profound and enduring effects on mental health. Evidence derived from the first wave of COVID-19 suggests that there is a widespread emotional distress linked to the COVID-19 pandemic [1–3].

The different epidemic control measures such as lockdown restrictions, schools and business closures, and social distancing have disrupted people’s daily lives, and the uncertainties/fears associated with the epidemic and the exceptional control measures have been linked to increases in anxiety and depression, meeting in many cases the threshold for clinical relevance [4–7]. In addition, concerns about fear of infection with COVID‐19, the consequences of infection for oneself or loved ones, and the financial instability have also contributed to the increase of anxiety and depressive symptoms [8]. Examining the impact of the pandemic on anxiety and depression is crucial since these disorders are accompanied by substantial disability and high recurrence rates [9–11]. Moreover, they often co-occur [12], and compared to having one disorder alone, this co-occurrence is associated with more severe psychopathology and a poorer clinical course [13, 14].

Previous evidence indicates that people with pre-pandemic mental disorders are more vulnerable to COVID-19-related stress (danger and contamination fears, fears about economic consequences, compulsive checking and reassurance seeking, traumatic stress symptoms about COVID-19) than the general population [15–18], and this may be linked to poorer coping abilities, disruptions to mental health care routines, jeopardizing of treatments, and the associated increases in the risk of relapse or exacerbation of symptoms [17, 19, 20].

On the other hand, social support is known to be a key protective factor for anxiety and depression [21, 22], and it may be particularly important to improve psychological wellbeing and to prevent mental disorders during times of crisis such as the COVID-19 pandemic [23]. For instance, a study with more than 700,000 college students showed that during the disease outbreak, individuals with low perceived social support were 4.8 and 6.0 times more likely to have anxiety and depressive symptoms, respectively, compared to individuals with high perceived social support [24]. Moreover, positive social support has shown to be protective against the risk for affective disorders by buffering the effects of stress and by enhancing coping strategies [25]. However, little is known regarding the moderating effects of pre-pandemic mental conditions on the association between social support and depression and anxiety in the context of COVID-19.

Therefore, the aim of this study is to identify the moderating effect of pre-pandemic mental disorders on the associations of COVID-related stress and social support with those screening positive for Generalized Anxiety Disorder (GAD) and Major Depressive Disorder (MDD), during the first wave of the COVID-19 pandemic. In addition, since anxiety and depression often co-occur [12], a secondary aim of the present study was to assess such associations in those screening positive for depression and anxiety (comorbid form).

## Methods

### Sample and study design

Data from a cross-sectional survey conducted in a nationally representative sample of the Spanish adult general population were analyzed. The eligible sample consisted of adults aged ≥ 18 years that had no language barriers to Spanish and had access to a mobile phone or landline telephone.

A bureau of professional interviewers conducted computer-assisted telephone interviews from June 1 to June 30, 2020. The sample was drawn using dual-frame random digit dialing, including both mobile (85%) and landline (15%) telephone numbers. First, a sample of Spanish mobile telephone numbers was generated via an automated system. Subsequently, landline numbers were selected from an internal database developed and maintained by the survey company to ensure that all Spanish geographical areas were adequately represented. Up to seven calls were attempted to each number. The sample distribution was planned according to quotas proportional to the Spanish population in terms of age groups, gender and region of residence (National Institute of Statistics in Spain, July 2019). A total of 138,656 numbers were sampled, with a final split of 71% mobile and 29% landline telephones. Of them, 45,002 were non-eligible (i.e., non-existing numbers, numbers of enterprises, numbers of people with Spanish language barriers, fax numbers and numbers belonging to quota that were already completed), and 72,428 had unknown eligibility (i.e., no contact was made after the seven attempted calls). Among the remaining 21.266 eligible numbers, 3500 agreed to participate in the interviews (cooperation rate of 16.5%).

Ethical approval was provided by the Fundació Sant Joan de Déu Ethics Committee, Barcelona, Spain (PIC 86-20) and by the Parc de Salut Mar Clinical Research Ethics Committee (2020/9203/I). Oral consent from all participants was obtained prior to proceeding with the interview.

### *Risk for screening positive for GAD, MDD and comorbid GAD* + *MDD (outcome variables)*

The Spanish versions of the 7-item Generalized Anxiety Disorder Scale (GAD-7) [26, 27] and the 8-item Patient Health Questionnaire Depression Scale (PHQ-8) [28–30] were employed to screen for GAD (outcome 1) and MDD (outcome 2), respectively. Both scales showed good internal consistencies in our sample (Cronbach’s *α* = 0.85 and 0.83, respectively). The recommended cut-off point of ≥ 10 was applied as a diagnostic threshold of GAD and MDD in corresponding scales [26–29]. To address the secondary aim, a dichotomized variable that included those participants screening positive in both scales (GAD-7 and PHQ-8 ≥ 10), versus those who had only depression, only anxiety or none was also created.

### COVID-related perceived stress and social support (predictors)

The COVID-related perceived stress was assessed with an adapted version of the Peri Life Events Scale [24, 25], that included 5 items “Concern about being probably infected by COVID-19”, “Concern regarding my loved ones being infected by COVID-19”, “Death of a loved one due to COVID-19”, “Job loss or income reduction due to COVID-19” and “Alarming or negative media reporting about COVID-19”. Each item was rated on a 5-point Likert scale ranging from (“none” to “very severe”). The total score was obtained by summing all responses, with higher scores reflecting greater levels of COVID-related perceived stress. (Cronbach’s *α* in the current sample was = 0.76).

The Oslo Social Support Scale (OSSS-3) [32] was used to assess social support. It has 3 items: “How many people are you so close to that you can count on them if you have great personal problems?” (“more than 5”, “from 3 to 5”, “from 1 to 2”, or “none”); “How much interest and concern do people show in what you do?” (“a lot”, “some”, “uncertain”, “little”, “none”), and “How easy is it to get practical help from neighbors if you should need it?” (“very easy”, “easy”, 3 “possible”, “difficult”, “very difficult”). The total score ranged from 3 to 14, with higher values representing higher levels of social support. (Cronbach’s *α* in current sample = 0.50).

### Pre-pandemic mental conditions

The presence of pre-pandemic lifetime mental disorders was assessed using a checklist based on the Composite International Diagnostic Interview (CIDI) that screens for self-reported lifetime depressive disorder, bipolar disorder, anxiety disorders, panic attacks, alcohol and drug use disorders, and “other” mental disorders [33]. A dichotomous variable (Y/N) was created (participants with ≥ 1 pre-pandemic, vs none).

### Covariates

Sociodemographic characteristics included age, gender, highest level of education (≤ primary, secondary, ≥ tertiary education), marital status (single, married, divorced/separated, widowed), and employment status (employed, unemployed, student, retired/sick-paid). Also, the number of rooms per person living in the household was calculated by dividing the house size (number of rooms) by the number of people living in it. Health-related covariates included positive diagnosis of the COVID-19 (Y/N) and the presence of chronic physical conditions assessed using 7-item checklists that included diabetes, cancer, cardiovascular diseases, chronic hepatic diseases, immunological diseases, respiratory diseases not caused by COVID-19, and “other” [34]. The answers to these questions were summed and the variable was operationalized as none, 1, and ≥ 2.

### Statistical analysis

To ensure sample representativeness and to compensate for potential survey non-response bias, all data were weighted with post-stratification weights to restore the distribution of the adult general population of Spain according to age groups, sex and geographic area. Missing survey data were minimal (median 0.17% [IQR 0.06–0.59%] across all survey variables) and addressed using fully conditional specification methods (FCS) [35]. Simulation studies provide evidence that FCS multiple imputation generally yields estimates that are unbiased and provide appropriate coverage, particularly under missing at random assumption [36]. Descriptive analyses were conducted to characterize the study sample. These analyses included unweighted frequencies and weighted proportions for categorical variables, and mean and standard deviations for continuous variables. The difference in sample characteristics by the three outcomes (GAD, MDD and comorbid GAD + MDD) was tested by Chi-squared tests for categorical variables and Student’s t-tests for continuous variables. Cronbach alpha coefficients were computed to estimate the internal-consistency reliability of each scale score. Unadjusted logistic regressions were fitted to test the relationships between pre-pandemic mental disorders, COVID-related stress, social support, and the remaining covariates with MDD, GAD and the comorbid form. Those variables that predicted the outcome (*p* < 0.20) were included in multivariate logistic regression models as covariates [37]. Interactions of pre-pandemic mental disorders with social support and COVID-related stress were tested in separate multivariate logistic regression models for GAD, MDD and the comorbid form as outcomes. Statistically significant interactions (*p* < 0.05) were included in the final multivariate regression models. To clarify statistically significant interaction effects, estimated probabilities of GAD and MDD were calculated based on the adjusted logistic regression models through margins command [38] adjusting for covariates at mean, taking the real proportion in the sample into account.

Results from the regression analyses are presented as odds ratios (ORs) with 95% confidence intervals (CIs). The level of statistical significance was set at *p* < 0.05. Statistical analyses were performed with Stata 14.1 (Stata Corp LP, College station, Texas).

## Results

A total of 3500 participants aged ≥ 18 years were included in the analysis. The mean age was 49.25 (SD = 15.64) and 51.50% were females. The prevalence of screening positive for GAD, MDD and the comorbid form (GAD + MDD) were 10.9%, 11.3%, and 6.63%, respectively. Among those without any pre-pandemic mental disorders, the prevalence of GAD, MDD and the comorbid form were 5.6%, 5.9%, and 2.96%. Among those with pre-pandemic mental disorders the prevalence of screening positive for GAD, MDD and the comorbid form were 21%, 22% and 13.67%, respectively. More information on the sample characteristics is provided in Table 1.

**Table 1:** Sample characteristics

	Overall (*n* = 3500)	Outcome 1 GAD yes (*n* = 395)	Outcome 2 MDD yes (*n* = 407)	Outcome 3 GAD + MDD yes (*n* = 242)
Age (years), *n (%)*				
18–34	697 (22.10)	111 (28.10)***	116 (16.18)***	62 (8.53)**
35–54	1501 (38.27)	180 (11.78)	177 (11.55)	112 (7.39)
55–64	680 (15.94)	63 (8.77)	71 (10.09)	43 (6.06)
+ 65	622 (23.68)	41 (6.62)	43 (6.93)	25 (4.02)
Gender, *n (%)*				
Male	1538 (48.50)	119 (7.88)	126 (8.35)	79 (5.15)
Female	1962 (51.50)	276 (13.75)***	281 (13.98)***	163 (8.04)***
Highest level of education, *n (%)*				
≤ Primary	237 (7.65)	40 (15.40)***	40 (15.91)***	27 (10.37)***
Secondary	1856 (52.7)	225 (11.78)	243 (12.52)	145 (7.44)
≥ Tertiary	1407 (39.5)	130 (8.87)	124 (8.65)	70 (4.84)
Marital status, *n (%)*				
Single	1180 (35.3)	161 (13.49)**	174 (14.51)***	99 (8.17)**
Married	1806 (49.7)	175 (9.08)	161 (8.40)	99 (5.14)
Divorced/separated	319 (8.2)	41 (11.86)	51 (14.96)	31 (9.02)
Widowed	195 (6.72)	18 (9.59)	21 (10.61)	13 (6.77)
Employment status, *n (%)*				
Employed	1788 (48.61)	187 (10.42)***	177 (9.89)***	98 (5.44)***
Unemployed	784 (21.67)	116 (14.34)	124 (15.41)	82 (10.20)
Student	131 (4.60)	22 (16.47)	24 (17.79)	13 (9.60)
Retired/sick-paid	719 (25.13)	58 (7.42)	63 (7.88)	39 (4.75)
Number of rooms per person, mean (SD)				
Per unit increase	1.38 (0.83)	1.24 (0.70)***	1.30 (0.79)	1.31 (0.75)
Infection status, *n (%)*				
Negative	3406 (94.46)	381 (10.83)	392 (11.14)	238 (6.72)
Positive	94 (2.54)	14 (13.62)	15 (15.41)	4 (3.34)
N. chronic physical conditions, *n (%)*				
None	2119 (60.30)	208 (9.55)***	208 (9.61)***	123 (5.66)***
1	997 (28.60)	110 (10.49)	122 (11.68)	64 (6.03)
> 1	384 (11.10)	77 (19.31)	77 (19.06)	55 (13.49)
Social support^a^, mean (SD)				
Per unit increase	11.13 (1.89)	10.57 (2.33)***	10.43 (2.39)***	10.35 (2.45)***
COVID-related perceived stress^b¸^ mean (SD)				
Per unit increase	13.19 (4.91)	16.38 (4.8)***	15.93 (4.88)***	16.68 (4.89)***
Pre-pandemic mental disorder, *n (%)*				
No	2274 (65.65)	134 (5.59)***	136 (5.87)***	70 (2.95)***
Yes	1226 (34.35)	261 (21.06)	271 (21.52)	172 (13.67)

Unweighted frequencies and weighted proportions are displayed for categorical variables. Mean and standard deviation (SD) are shown for continuous variables. Percentages in the overall column show the distribution of the sample, while GAD, MDD and GAD+MDD columns show proportion by sample characteristics. Asterisks reflect difference in sample characteristics by GAD, MDD and GAD+MDD (yes vs no) as indicated by Chi- and Student’s *t*-tests. **p < 0.01, ***p < 0.001

*GAD* generalized anxiety disorder, *MDD* major depressive disorder, *COVID* coronavirus infectious disease

^a^Scores ranged from 3 to 14 with higher scores representing higher levels of social support

^b^Scores ranged from 5 to 25 with higher scores representing higher levels of perceived stress

Bivariate logistic regression models showed that higher levels of COVID-related stress and having a pre-pandemic mental disorder were significantly associated with increased risk of screening positive for GAD, MDD and comorbid GAD + MDD (for COVID-related stress, OR ranging from 1.14 to1.18, *p* < 0.001; and OR ranging from 4.40 to 5.20, *p* < 0.001 for pre-pandemic mental disorders). Higher levels of social support were related to lower odds of screening positive for GAD, MDD and comorbid GAD + MDD (OR 0.81–0.85, *p* < 0.001). Additionally, being female, being unemployed or a student, and reporting ≥ 2 chronic somatic conditions were common significant risk factors. On the other hand, being older, a higher educational level, and being married were significantly associated with a lower risk of for screening positive on GAD, MDD and comorbid GAD + MDD (Table 2).

**Table 2:** Unadjusted logistic regression models of factors related to GAD, MDD and the comorbid form

Characteristic	Outcome 1 GAD	(95% CI)	Outcome 2 MDD	(95% CI)	Outcome 3 GAD + MDD	
	OR		OR		OR	(95% CI)
Age						
18–34	Ref		Ref		Ref	
35–54	0.73*	(0.56, 0.94)	0.68**	(0.52, 0.87)	0.86	(0.61, 1.19)
55–64	0.52***	(0.38, 0.73)	0.58**	(0.42, 0.80)	0.69	(0.46, 1.04)
+ 65	0.39***	(0.26, 0.56)	0.39***	(0.27, 0.56)	0.50**	(0.28, 0.73)
Gender						
Female vs male	1.84***	(1.48, 2.35)	1.78***	(1.42, 2.24)	1.61**	(1.21, 2.13)
Highest level of education						
≤ Primary	Ref		Ref		Ref	
Secondary	0.73	(0.50, 1.07)	0.76	(0.51, 1.11)	0.69	(0.44, 1.09)
≥ Tertiary	0.53**	(0.36, 0.80)	0.50**	(0.34, 0.75)	0.44**	(0.27, 0.71)
Marital status						
Single	Ref		Ref		Ref	
Married	0.64***	(0.51, 0.81)	0.54***	(0.43, 0.68)	0.61**	(0.45, 0.82)
Divorced/separated	0.86	(0.59, 1.26)	1.04	(0.73, 1.50)	1.11	(0.72, 1.72)
Widowed	0.68	(0.41, 1.14)	0.70	(0.43, 1.14)	0.82	(0.44, 1.50)
Employment status						
Employed	Ref		Ref		Ref	
Unemployed	1.43**	(1.12, 1.86)	1.66***	(1.29, 2.14)	1.97***	(1.44, 2.70)
Student	1.70*	(1.04, 2.76)	1.97**	(1.22, 3.17)	1.84	(1.00, 3.42)
Retired/sick-paid	0.69*	(0.50, 0.95)	0.78	(0.57, 1.06)	0.87	(0.58, 1.29)
Number of rooms per person						
Per unit increase	0.77**	(0.65, 0.91)	0.87	(0.75, 1.01)	0.89	(0.74, 1.06)
Infection status						
Positive vs negative	1.30	(0.72, 2.35)	1.45	(0.81, 2.60)	0.49	(0.18, 1.35)
N. of chronic physical conditions						
None	Ref		Ref		Ref	
1	1.11	(0.86, 1.43)	1.24	(0.97, 1.59)	1.07	(0.78, 1.47)
> 1	2.27***	(1.68, 3.05)	2.22***	(1.64, 2.98)	2.60***	(1.83, 3.68)
Social support						
Per unit increase	0.85***	(0.80, 0.90)	0.81***	(0.77, 0.86)	0.81***	(0.75, 0.87)
COVID-related perceived stress						
Per unit increase	1.17***	(1.14, 1.20)	1.14***	(1.12, 1.17)	1.18***	(1.14, 1.21)
Pre-pandemic mental disorder						
Yes vs no	4.51***	(3.59, 5.67)	4.40***	(3.51, 5.51)	5.20***	(3.87, 6.99)

*GAD* generalized anxiety disorder, *MDD* major depressive disorder, *OR* odds ratio, *COVID* coronavirus infectious disease, *CI* confidence interval

^*^p < 0.05, **p < 0.01, ***p < 0.001

Results of the significant interaction effects between pre-pandemic mental disorders, COVID-related stress and social support for GAD, MDD and comorbid GAD + MDD are shown in Table 3. Pre-pandemic mental disorders significantly moderated the relationship with COVID-related stress for GAD and MDD, whereas pre-pandemic mental disorders significantly moderated the relationship between social support and GAD, but not MDD [OR 95% CI 1.00 (0.87–1.13)]. As for the secondary analysis, no significant interactions were found for comorbid GAD and MDD either for COVID-related stress or social support [OR 95% CI 0.95 (0.88–1.02); 1.09 (0.93–1.28), respectively].

**Table 3:** Adjusted logistic regression models of factors related to the three outcomes (GAD, MDD and the comorbid form)

	(Model 1) GAD OR (95% CI)	(Model 2) MDD OR (95% CI)	(Model 3) GAD + MDD OR (95% CI)
Social support	0.80 (0.72, 0.88)***	0.84 (0.79, 0.89)***	0.85 (0.79, 0.91)***
COVID-related stress	1.18 (1.13, 1.23)***	1.16 (1.11, 1.20)***	1.15 (1.11, 1.19)***
Pre-pandemic mental disorder	1.59 (0.31, 8.29)	9.65 (4.15, 22.41)***	3.47 (2.52, 4.79)***
Interactions			
Social support * pre-pandemic mental disorder	1.18 (1.04, 1.34)*	–	–
COVID-related stress * pre-pandemic mental disorder	0.94 (0.89, 0.99)*	0.93 (0.88, 0.97)**	–

Models included all variables, significant interaction terms shown in the table and age, gender, highest level of education, marital status, employment status, number of rooms per person, and number of chronic physical conditions.

*COVID* coronavirus infectious disease, *CI* confidence interval, *GAD* generalized anxiety disorder, *MDD* major depressive disorder

*p  <  0.05, **p  < 0.01, ***p  ≤  0.001

**Fig. 1:**
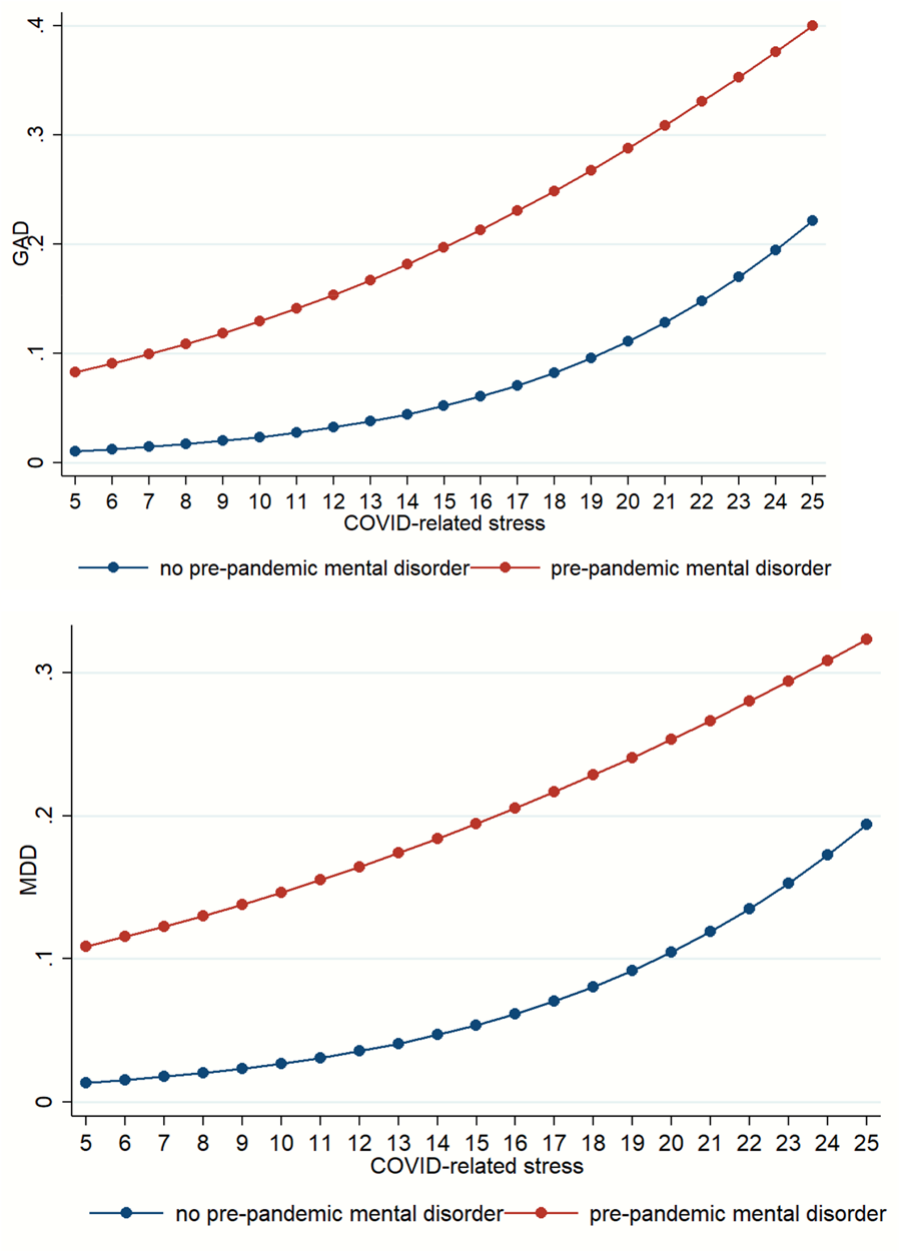
Probabilities for GAD and MDD according to pre-pandemic mental disorders and COVID-related stress. Estimated probabilities of GAD and MDD were calculated based on the adjusted logistic regression models through margins command [38] adjusting for covariates at mean, taking into account the real proportion in the sample. GAD: generalized anxiety disorder; MDD: major depression disorder, COVID: coronavirus infectious disease

**Fig. 2:**
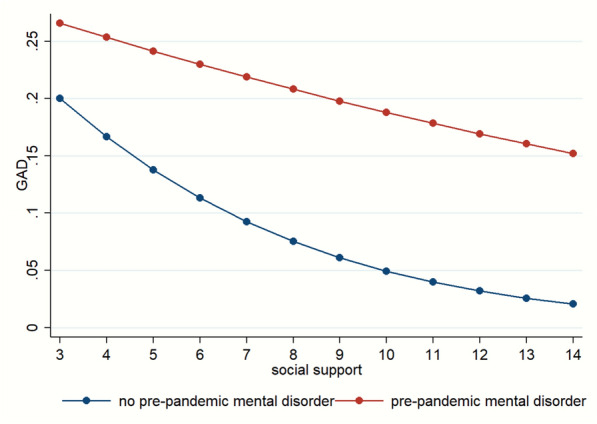
Probabilities for GAD by pre-pandemic mental disorders and social support according to logistic regression model. Estimated probabilities of GAD and MDD were calculated based on the adjusted logistic regression models through margins command [38] adjusting for covariates at mean, taking the real proportion in the sample into account. GAD: generalized anxiety disorder; MDD: major depression disorder

Patterns of significant interactions between pre-pandemic mental disorders and COVID-related stress for both GAD and MDD indicated that higher COVID-related stress predicted increased risk of GAD and MDD in both groups (Fig. 1). The pattern of interaction between pre-pandemic mental disorders and social support for GAD showed that among those who had no pre-pandemic mental disorder, higher levels of social support decreased the probabilities of screening positive for GAD, while among those who had a pre-pandemic mental condition, higher levels of social support had very modest decreases in the risk of GAD (Fig. 2).

## Discussion

Our study provides an extension of previous evidence by examining the link between COVID-19-related stress and social support with the risk of GAD, MDD and comorbid GAD + MDD. Furthermore, we provided evidence on the moderating effect of pre-pandemic mental conditions in the association between COVID-19-related stress and social support with GAD, MDD and comorbid GAD + MDD.

The present study found that higher COVID-related stress predicted increased risk of GAD, MDD and comorbid GAD + MDD, which is consistent with previous COVID-based evidence [15, 39]. While pre-pandemic mental disorders did not significantly moderated the relationship with COVID-related stress and comorbid GAD + MDD, significant interactions were found for only GAD and only MDD. Similar patterns according to pre-pandemic mental disorders were found, but those with pre-pandemic mental conditions have a higher risk and more steady increases in the odds of screening positive these affective disorders.

These findings are consistent with previous studies demonstrating that people with a pre-pandemic mental health disorder are more negatively impacted by COVID-related stress [15–17], which is correlated with an increased likelihood to be concurrently depressed or anxious [15]. The increased susceptibility of people with pre-pandemic mental disorders to COVID-related stressors might be caused by different factors including the disruption of daily routines and mental health care caused by lockdown and mobility restrictions [19, 20], and a higher difficulty to cope with the COVID-19 pandemic [17]. However, there is conflicting evidence regarding whether there has been an increase in symptoms during the pandemic. For instance, a recent systematic review shows that people with pre-pandemic mental disorders have significantly higher psychiatric symptoms, anxiety symptoms and depressive symptoms compared to controls during a pandemic [40], while others do not report such increases [17]. Future research is warranted to examine the mental health impact and coping mechanisms of those with and without pre-pandemic mental conditions during the COVID-19 pandemic in the medium and long term.

With regard to social support, we found that higher levels of social support were related to lower risk of GAD, MDD and the comorbid form, which is consistent with previous evidence conducted in the general population during the first wave of the COVID-19 [41–43]. Social support contributes to coping with traumatic experiences and it is important for buffering individual psychological responses to life crises [44, 45]. Although the exact pathways through which perceived social support operates to reduce the risk of mental disorders are unclear, several mechanisms have been suggested. First, social support acts as a buffer against the negative impact of COVID-related stressors [39], possibly through the promotion of feelings of security and sense of control over the situation, which may enhance self-esteem and therefore reduce the impact of stress on the psychological adjustment [46, 47]. Additionally, social support may provide protection form stressful events and reduce the affective reaction by attenuating or preventing a stress appraisal [48], by providing distraction from the problem, preventing maladaptive behavioral responses [49] and facilitating health promotion behaviors, factors that may ultimately act on regulating physiological processes [25].

The presence of pre-pandemic mental disorders did not moderate the relationship between social support and MDD and comorbid GAD + MMD, but a significant interaction was found for GAD. That is, higher social support levels were a protective factor for those with and without pre-pandemic mental condition, but for those with a pre-pandemic mental condition, higher levels of social support were related to very modest decreases in the risk of GAD. It is possible that for those with pre-pandemic mental conditions, other protective/risk factors may be more relevant for preventing the risk of GAD during the COVID-19 outbreak or that they need first to manage their distress or symptomatology before being able to benefit from social support. It is also possible that for people with pre-pandemic disorders it may be more difficult to draw support from their social circles [50] and to socially withdraw, which may ultimately enhance the feelings of loneliness and subsequently the risk for current GAD. Further longitudinal research is needed in order to better understand the role of pre-pandemic mental conditions in the association between social support and affective disorders in the context of a health crisis. Additionally, digital interventions for vulnerable population groups at risk of worse mental health linked to the COVID-19 are increasing (e.g., those under being infected, and under quarantine) [51, 52], and telehealth and digital interventions could be a useful tool to improve social support and to guarantee that people with pre-pandemic mental disorders have access to their psychological interventions and treatment during a pandemic such as COVID-19 or when social distancing measures are established [53].

The study results should be interpreted in light of several limitations. First, the cross-sectional nature of the study does not allow to infer causal conclusions. Also, given that the course of the pandemic is uncertain, longitudinal evidence assessing multiple time points is warranted to better understand the impact of COVID-19 in population’s mental health, and to disentangle the exact contribution of correlates of risk and protection. Second, while the sample is representative of the general population, present findings cannot be generalized to other institutionalized populations as well as other hard to reach groups. Third, the assessment of mental disorders was based on self-reported screening scales (GAD and MDD) and a CIDI checklist (pre-pandemic lifetime disorders), but these assessments are inferior to face-to-face clinical assessments. Forth, in our sample, the OSSS-3 had a lower internal consistency coefficient compared to previous studies (*Cronbach’s* *α* = 0.50 versus 0.64) [32]. A possible reason for this lower value might be due to the use of this scale in the context of COVID-19, in which social relationships were directly affected and for which the scale had not been previously validated. Finally, given the relatively modest sample size of the comorbid MDD + GAD group, it is possible that null results in the interaction analysis for those scoring positive on comorbid GAD + MDD might be underpowered due to the small sample size.

## Conclusions

In conclusion, current findings suggest that higher COVID-related stress predicted increased risk of GAD, MDD and comorbid GAD + MDD, with potential greater adverse consequences for those with pre-pandemic mental disorders. In addition, interventions focused on increasing social support to manage psychological distress may be effective in reducing affective disorders, independently of the presence of a pre-pandemic disorder.

## Data Availability

The de-identified participant data as well as the study protocol, statistical analysis plan, and data dictionaries used for this study are available as from publication and upon reasonable request from the corresponding author as long as the main objective of the data sharing request is replicating the analysis and findings as reported in this paper (without investigator support), after approval of a proposal, and with a signed data access agreement.

## Abbreviations

SARS-Cov-2: Severe acute respiratory syndrome coronavirus 2
COVID-19: Coronavirus infectious disease 19
GAD: Generalized anxiety disorder
MDD: Major depressive disorder
GAD-7: 7-Item Generalized Anxiety Disorder Scale
PHQ-8: 8-Item Patient Health Questionnaire Depression Scale
OSSS-3: 3-Item Oslo Social Support Scale
CIDI: Composite International Diagnostic Interview
FCS: Fully conditional specification methods
OR: Odds ratio
CI: Confidence interval
